# Assessment of Functional Outcome and Postoperative Complications in Proximal Humerus Fracture Patients Managed With Proximal Humerus Internal Locking System (PHILOS) Plating

**DOI:** 10.7759/cureus.63250

**Published:** 2024-06-26

**Authors:** Udit Agrawal, Vaibhav B K., Harsh Kirthi Rao, Praseeth K R., Durga K Narayandas

**Affiliations:** 1 Pediatric Orthopedics, King George's Medical University, Lucknow, IND; 2 Orthopedics and Traumatology, Srinivas Institute of Medical Sciences and Research Center, Mangalore, IND

**Keywords:** philos plating, prospective study, complications, functional outcome, proximal humerus fracture

## Abstract

Background: Proximal humerus fractures are primarily common in the old age group. The appropriate approach to managing such displaced and comminuted fracture patterns is often questionable. Hence, this study was conducted to assess the functional outcome of proximal humerus fractures following treatment with a proximal humerus locking plate and to assess the frequency of complications in such patients.

Methodology: In this robust study, 33 cases of proximal humerus fractures underwent surgical management at a prestigious teaching hospital from February 2021 to August 2022 utilizing a proximal humerus internal locking system (PHILOS) plate. The NEER classification was employed to categorize the fractures, and the NEER score was used for functional assessment. It's crucial to note that individuals with pathological fractures, associated injuries in the ipsilateral limb, nerve injuries, and cases of open fracture were rigorously excluded from the study.

Results: The mean age was 47 ± 5.2 years. Based on NEER's classification, the distribution of fractures was as follows: two-part fractures accounted for 18.18% (n = 6) of cases, three-part fractures for 54.54% (n = 18) of cases, and four-part fractures for 27.27% (n = 9) of cases. A history of road traffic accidents and falls was reported in 54.54% (n = 18) and 45.45% (n = 15) of cases, respectively. Functional outcome assessment utilizing NEER's score revealed a minimum score of 48 and a maximum of 96, with an average score of 82.96 ± 12.73. Notably, 39% of patients demonstrated excellent results, 27% exhibited satisfactorily, 21% manifested unsatisfactorily, and 12.12% presented failure outcomes. Of the 33 operated cases, 81.8% (n = 27) exhibited no complications during follow-up. The predominant complication observed was shoulder stiffness (9.09%, n = 3), followed by Varus mal-union (6.06%, n = 2), and superficial surgical site infection (3.03%, n = 1), managed with debridement and antibiotics leading to subsequent resolution.

Conclusions: Managing proximal humerus fractures has consistently posed a formidable challenge. Our study indicates that using the PHILOS plate represents a reliable option for addressing such fractures. This plate provides sturdy fixation, facilitates early mobilization, and culminates in exceptional functional outcomes. The insights gained from this study can inform clinical decision-making and guide orthopedic surgeons in selecting the appropriate treatment strategy for proximal humerus fracture patients.

## Introduction

Fractures of the proximal humerus constitute 4% to 6% of all humerus fractures and 25% of shoulder fractures [1]. Managing unstable fractures presents a significant challenge due to factors including osteoporotic bone, angular instability, hardware prominence, loss of reduction, and screw back out [2]. In contrast to the more prevalent occurrences of indirect trauma observed in older people, younger individuals are at higher risk of experiencing high-energy trauma. The debate between conservative and surgical interventions remains contentious, with each approach offering distinct advantages and disadvantages [3]. The primary aim of treatment should be the restoration of a pain-free shoulder and the resumption of normal daily activities [4].

Hertel et al. conducted a perfusion test on proximal humerus fractures to assess humeral head ischemia. Their findings underscore the importance of metaphyseal head extension in these fractures. Notably, a metaphyseal head extension of less than 8 mm proved to be a robust indicator of ischemia [5]. Furthermore, a medial hinge exceeding 2 mm emerged as another reliable predictor of ischemia [6]. Progressive dislocation may ensue as a consequence of non-operative management, attributed to the unopposed traction exerted by the rotator cuff muscles. Consequently, the non-operative management of such dislocated fractures has become the topic of increasing discourse. Non-operative intervention may give rise to complications, including non-union, osteonecrosis, and malunion. Consequently, operative management is deemed imperative in most cases [3].

Over the past decade, there has been a remarkable advancement in the range of implants accessible to address such injuries. Fixation techniques for these fractures encompass K-wires, cerclage wires, bone sutures, tension band wires, T-plates, intramedullary devices, and prosthetic replacements [7]. In the elderly population, the humeral head cancellous bone stock is notably diminished, predisposing conventional plates to implant failure. The newly developed locking plates are meticulously tailored for proximal humeral fixation, with careful consideration given to the anatomical characteristics of this region [8]. From a biomechanical standpoint, these implants exhibit comparatively lower stiffness, and the design of their locking screw head ensures unimpeded periosteal blood flow, thus rendering them particularly suitable for osteoporotic bone [9].

The Proximal Humerus Internal Locking System (PHILOS) stands as a pioneering anatomical locking plate developed by the American Orthopaedic Foundation and Orthopaedic Trauma Association (AO/OTA) with the specific aim of maximizing functional outcomes, particularly for elderly patients. What truly sets PHILOS apart is its exceptional ability to deliver angled stabilization through the use of multiple interlocking screws, establishing it as a superior choice over traditional plates [10]. The superior locking mechanism of the screws significantly enhances bone anchorage, provides robust angular stability, preserves postoperative reduction, prevents joint stiffness, and decisively improves functional outcomes [11]. This study aims to assess the functional outcome and postoperative complications in proximal humerus fracture patients managed with PHILOS plating.

## Materials and methods

This prospective study involved 33 patients who presented to the emergency department of KVG Medical College and Hospital, Sullia, India, between February 2021 and August 2022 with closed proximal humerus fractures. These patients were managed with PHILOS plating. The study received approval from the Institutional Ethics Committee of Rajiv Gandhi University before its commencement (approval letter no. IEC/RGU/ACA/DCD/SYN/KVG-SPG/2021).

The inclusion criteria encompassed patients aged 18 and above with two-, three-, or four-part proximal humerus fractures displaying a displacement of >1 cm and a varus angulation of >45 degrees who underwent operative management with a proximal humerus locking plate and expressed willingness to participate in the study. The study's exclusion criteria encompassed individuals with pathological fractures, associated injuries in the ipsilateral limb, major nerve injuries (e.g., axillary nerve or deltoid palsy), and cases of open fracture.

Upon arrival at the emergency room, all patients with a history of shoulder injury underwent a comprehensive examination. Analgesics were administered for pain relief, and standard anteroposterior and Y-view radiographs of the shoulder joint were prescribed to assess the nature of the fracture. A U-shaped slab and an arm pouch were applied to provide immobilization. Fractures were classified according to the NEER classification [12], and routine pre-anesthetic investigations were conducted. After the pre-anesthetic evaluation, the patient's suitability for surgery was determined, and informed written consent was obtained. As part of the treatment, patients underwent open reduction internal fixation with a PHILOS plate using a standard deltopectoral approach under general anesthesia.

Patients were consistently monitored at 2, 6, 12, and 24 weeks following the procedure. Standard anteroposterior and Y-view radiographs of the shoulder joint were recommended to evaluate fracture union and postoperative complications. Functional outcomes were assessed using NEER's score [13] at the 24-week follow-up. Patients were stratified into categories of excellent (90-100), satisfactory (80-89), unsatisfactory (70-79), and failure (<70) based on their scores.

Postoperative rehabilitation commenced according to the established protocol. This protocol involved initiating Pendulum exercises immediately postoperatively based on pain tolerance. Passive range of motion exercises were initiated in the first week, while active range of motion exercises began between two and four weeks postoperatively, contingent upon osteosynthesis stability and bone quality. Active assisted movements were introduced, limiting abduction to 90 degrees with no forced external rotation. Subsequently, a full range of movements with active exercises was initiated between the sixth and eighth week.

The statistical analysis was conducted using IBM SPSS Statistics for Windows, Version 25 (Released 2017; IBM Corp., Armonk, New York, United States). Descriptive statistics were utilized to compute and present frequencies and percentages. Mean and standard deviation (SD) were employed to assess central tendency and dispersion for quantitative variables.

## Results

This study analyzed 33 cases of proximal humerus fractures that underwent surgical management using the PHILOS plating. The average age of the cohort was 47 ± 5.2 years, with the highest proportion falling within the 41-59 years age bracket (45.45%, n = 15). Males represented 54.54% (n = 18) of the cases. Notably, fractures were predominantly located on the non-dominant left side (63.63%, n = 21) as opposed to the dominant right side. The mean duration from injury to the procedure was 4.36 ± 1.66 days (Table 1).

**Table 1:** Baseline characteristics of patients

Gender distribution	
Male	18 (54.54%)
Female	15 (45.45%)
Age Distribution	
21-30 years	5 (15.15%)
31-40 years	8 (24.24%)
41-50 years	3 (9.09%)
51-60 years	12 (36.36%)
>60 years	5 (15.15%)
Side affected	
Left	21 (63.63%)
Right	12 (36.36%)
Duration from Injury to surgery	
Average duration	4.36 ± 1.66 days
Time to bony union	
Average duration	13.57 ± 1.71 weeks

Based on NEER's classification, the distribution of fractures was as follows: two-part fractures accounted for 18.18% (n = 6) of cases, three-part fractures for 54.54% (n = 18) of cases, and four-part fractures for 27.27% (n = 9) of cases. A history of road traffic accidents (RTAs) and falls were reported in 54.54% (n = 18) and 45.45% (n = 15) of cases, respectively (Table 2).

**Table 2:** Patient distribution as per the mode of injury and type of fracture RTA: road traffic accident

NEER classification	Mode of injury		Total patients
	RTA	FALL	
2 Part (NEER type 2)	4 (12.12%)	2 (6.06%)	6 (18.18%)
3 Part (NEER type 3)	10 (30.30%)	8 (24.24%)	18 (54.54%)
4 Part (NEER type 4)	4 (12.12%)	5 (15.15%)	9 (27.27%)
Total	18 (54.54%)	15 (45.45%)	33 (100%)

Functional outcome assessment utilizing NEER's score (13) revealed a minimum score of 48 and a maximum of 96, with an average score of 82.96 ± 12.73. Notably, 39% of patients demonstrated excellent results, 27% exhibited satisfactorily, 21% manifested unsatisfactorily, and 12.12% presented failure outcomes (Table 3). The mean duration for fracture union was 13.57 ± 1.71 weeks, with 45.45% of patients achieving union at 12 weeks, 33.33% at 14 weeks, 18.18% at 16 weeks, and 3.03% at 18 weeks.

**Table 3:** Patient distribution as per functional outcome (NEER score) against the type of fracture

Grading	Type of fracture		
	Two-part (n = 6)	Three-part (n = 18)	Four-part (n = 9)
Excellent	5 (15.15%)	7 (21.21%)	1 (3.03%)
Satisfactory	1 (3.03%)	7 (21.21%)	1 (3.03%)
Unsatisfactory	0 (0%)	3 (9.09%)	4 (12.12%)
Failure	0 (0%)	1 (3.03%)	3 (9.09%)

Of the 33 operated cases, 81.8% (n = 27) exhibited no complications during follow-up. The predominant complication observed was shoulder stiffness (9.09%, n = 3), followed by Varus mal-union (6.06%, n = 2) and superficial surgical site infection (3.03%, n = 1), which were managed with debridement and antibiotics and led to subsequent resolution (Table 4). Furthermore, the study did not evidence proximal humeral osteonecrosis or non-union. Notably, shoulder stiffness improved with physiotherapy.

**Table 4:** Patient distribution as per postoperative complications

Postoperative complication	No. of patients
No complication	27 (81.81%)
Stiffness	3 (9.09%)
Varus malunion	2 (6.06%)
Superficial infection	1 (3.03%)

## Discussion

Fractures of the proximal humerus pose treatment challenges. They are seen in both young and older age groups, with younger people experiencing high-energy velocity injuries and older individuals having minor traumas. Undisplaced fractures can be managed non-operatively, but those with intra-articular extension and severe comminution require surgical fixation [14]. The anatomical location of these fractures renders conservative measures, such as bracing, ineffective [15].

Surgical modalities, such as percutaneous K-wires, offer advantages such as reduced soft tissue damage, minimized blood loss, and lowered risk of neurovascular injury. However, these techniques may not consistently deliver stable anatomical reduction and can impede early mobilization and fracture healing [16]. Furthermore, complications, including pin tract infection and delayed mobilization, limit the indications for this approach [4].

Internal fixation utilizing non-locking plates has historically yielded suboptimal clinical outcomes and elevated failure rates. Conversely, pre-contoured anatomical locking compression plates offer enhanced versatility and superior union rates, particularly in osteoporotic bones [17]. These plates afford more excellent lateral buttress stability, and their diverging screw options in cancellous bone render them the implant of choice for complex fractures. Forces are conveyed from the bone to the screw head and subsequently to the plate, endowing these plates with superior stability compared to non-locking plates [18]. A cadaveric study by Siffri et al. demonstrated that locking plates offer significantly better torsional stability compared to non-locking plates [19].

In our study, the average age was 47 ± 5.2 years, mirroring the age distribution noted in the work of Egol et al. [20] and Doshi et al. [10]. Most individuals (45.45%, n = 15) fell within the 41-59 age bracket. Out of 33 cases, 15 (45.45%) participants had experienced a fall, while 18 (54.54%) had a history of RTAs. The results align with previous research conducted by Kirsch and colleagues [21], wherein 47.5% of the 40 evaluated cases involved traffic accidents, and 50% reported falling incidents.

As per the NEER's classification, two-part fractures accounted for 18.18% (n = 6) of cases, three-part fractures for 54.54% (n = 18) of cases, and four-part fractures for 27.27% (n = 9) of cases. Yadav et al. [3] conducted a study demonstrating similar outcomes in the surgical management of 44 patients presenting with fresh three- and four-part proximal humerus fractures. In 21 patients, the PHILOS system was used for open reduction and internal fixation, while in 23 patients, k-wires were used for closed reduction and internal fixation.

Our study using NEER's score showed that 39% of patients displayed excellent results, 27% exhibited satisfactorily, 21% showed unsatisfactory results, and four patients presented failure outcomes. These findings align with research by Doshi et al. [10], which indicated that 13.21% of cases yielded excellent results, 69.81% were classified as satisfactory, 11.32% were deemed unsatisfactory, and 5.66% resulted in failure outcomes.

In our study, the predominant complication identified was shoulder stiffness, present in 9.09% (n = 3) of cases, followed by varus malunion in 6.06% (n = 2). These findings are consistent with the results reported in a study by Jhamnani et al. [3], who mentioned stiffness and malunion in 9.38% of cases each.

This study has several limitations. First, it was carried out with a relatively small sample size and was confined to a single center. Second, the follow-up duration was limited. Third, it lacked a comparison group.

## Conclusions

The management of proximal humerus fractures has consistently posed a formidable challenge. Our study underscores the significance of the PHILOS plating technique in achieving favorable functional outcomes and minimizing postoperative complications in proximal humerus fracture patients. The insights gained from this study can inform clinical decision-making, guiding orthopedic surgeons in selecting the most appropriate treatment strategy for proximal humerus fracture patients. However, studies with a larger sample size, a longer follow-up, and a comparable group will be needed to substantiate this statement.
